# Isotropy of Angular Frequencies and Weak Chimeras with Broken Symmetry

**DOI:** 10.1007/s00332-016-9345-2

**Published:** 2016-11-10

**Authors:** Christian Bick

**Affiliations:** Centre for Systems, Dynamics and Control and Department of Mathematics, University of Exeter, Exeter, EX4 4QF UK

**Keywords:** Oscillator networks, Phase oscillators, Weak chimera, Symmetry, Asymptotic average frequencies, 34C15, 37E45, 37C80

## Abstract

The notion of a weak chimeras provides a tractable definition for chimera states in networks of finitely many phase oscillators. Here, we generalize the definition of a weak chimera to a more general class of equivariant dynamical systems by characterizing solutions in terms of the isotropy of their angular frequency vector—for coupled phase oscillators the angular frequency vector is given by the average of the vector field along a trajectory. Symmetries of solutions automatically imply angular frequency synchronization. We show that the presence of such symmetries is not necessary by giving a result for the existence of weak chimeras without instantaneous or setwise symmetries for coupled phase oscillators. Moreover, we construct a coupling function that gives rise to chaotic weak chimeras without symmetry in weakly coupled populations of phase oscillators with generalized coupling.

## Introduction

The emergence of collective dynamics in networks of coupled oscillatory units is a fascinating phenomenon observed in science and technology (Strogatz 2000; Pikovsky et al. 2003). Symmetric phase oscillator networks provide paradigmatic models to understand collective dynamics in the weak coupling limit (Strogatz 2004; Acebrón et al. 2005; Tchistiakov 1996; Ashwin et al. 2016c). Such dynamical systems with symmetry are equivariant with respect to the action of a group (Golubitsky et al. 1988; Golubitsky and Stewart 2002; Field 2007), that is, the vector field commutes with the group action on phase space. Equivariance implies that any solution of the system is mapped to another solution by the action of the symmetry group and it typically constrains the dynamics, for example, by giving rise to dynamically invariant subspaces. The solutions themselves may (but do not have to) have nontrivial symmetry, that is, there may be nontrivial elements elements of the symmetry group that keep the solution fixed, either pointwise or as a set. For example, for globally coupled identical oscillators, the solution corresponding to full synchrony, where the states of all oscillators are equal, has full symmetry itself. Of course, there may be other solution with less symmetry relative to the symmetries of the system.

Recently, the observation of “symmetry breaking” in symmetrically coupled phase oscillator systems, i.e., the observation of solutions with localized synchronous dynamics coexisting with localized incoherence, has sparked a lot of interest. Such solutions, commonly known as chimera states—see Panaggio and Abrams (2015) for a recent review—were first observed in symmetric rings of coupled phase oscillators (Kuramoto and Battogtokh 2002; Abrams and Strogatz 2004). In the limit of infinitely many oscillators, they correspond to stationary or periodic patterns of the phase density distribution (Abrams et al. 2008; Omel’chenko 2013). By contrast, it was not until recently that Ashwin and Burylko (2015) gave a testable mathematical definition for chimera states, a *weak chimera*, for networks of finitely many phase oscillators whose phases $\varphi _k\in {\mathbf {T}}={\mathbb {R}}/2\pi {\mathbb {Z}}$, $k=1, \cdots , n$, evolve according to

1 $$\begin{aligned} \frac{{\mathrm {d}}\varphi _k}{{\mathrm {d}}t} = \dot{\varphi }_k = \omega + \frac{1}{n} \sum _{j=1}^n H_{kj} g(\varphi _k-\varphi _j). \end{aligned}$$

Here, the $H_{kj}$ determine the network topology (respecting a subgroup $\Gamma$ of the group ${\mathbf {S}}_n$ of permutations of $n$ symbols acting transitively on the indices of the oscillators) and $g{:}{\mathbf {T}}\rightarrow {\mathbb {R}}$ is the generalized coupling (or phase interaction) function. Weak chimeras are defined in terms of partial angular frequency synchronization on trajectories. More precisely, if $\hat{\varphi }$ is a continuous lift of a solution $\varphi$ of (1) with initial condition $\varphi ^0$ to ${\mathbb {R}}^n$ define the asymptotic angular frequency of oscillator *k* as

2 $$\begin{aligned} \Omega _k\left( \varphi ^0\right) = \lim _{T\rightarrow \infty }\frac{\hat{\varphi }(T)}{T}. \end{aligned}$$

According to Ashwin and Burylko (2015), a compact, connected, chain-recurrent, and dynamically invariant set $A\subset {\mathbf {T}}^n$ is a weak chimera if there are distinct oscillators $j,k,\ell$ such that $\Omega _j\left( \varphi ^0\right) =\Omega _k\left( \varphi ^0\right) \ne \Omega _\ell \left( \varphi ^0\right)$ for all $\varphi ^0\in A$.

Weak chimeras and angular frequency synchronization relate to symmetry. Assuming that all limits (2) exist, we have a frequency vector

$$\begin{aligned} \Omega \left( \varphi ^0\right) =\left( \Omega _1\left( \varphi ^0\right) , \cdots , \Omega _n\left( \varphi ^0\right) \right) \in {\mathbb {R}}^n. \end{aligned}$$

The group ${\mathbf {S}}_n$ also acts on ${\mathbb {R}}^n$ by permuting indices. If *A* is a weak chimera as above and $\tau _{kj}\in {\mathbf {S}}_n$ denotes the transposition swapping indices *k* and *j*, then $\tau _{kj}\Omega \left( \varphi ^0\right) =\Omega \left( \varphi ^0\right)$. That is, $\tau _{kj}$ is a symmetry of the angular frequency vector $\Omega \left( \varphi ^0\right)$. While weak chimeras have provided a suitable framework to derive for example existence results (Bick and Ashwin 2016), there are two shortcomings. First, while chimera states have also been reported in more general oscillator models (Sethia et al. 2013; Zakharova et al. 2014), the definition above applies to phase oscillators only. Second, the symmetries of the angular frequency vector $\Omega$ may be different from the symmetries of the system. As a consequence, if *A* is a weak chimera, then $\tau _{kj}A$ may not be a weak chimera or even a solution of the system at all. Interestingly, while it has been argued that chimera states are relevant due to their nature of solutions with broken symmetry (Abrams and Strogatz 2004), their properties have never been phrased in terms of symmetries of the dynamical system.

The contribution of this paper is twofold: First, we give a definition of a weak chimera in the language of equivariant dynamical systems and, second, we show that symmetries of the solution are not necessary for the occurrence of weak chimeras. More precisely, we define weak chimeras in terms of the isotropy of the angular frequency vector which can be stated for more general oscillator systems. We observe that, in a suitable setup, asymptotic angular frequencies are averages of equivariant observables. Therefore, symmetries of solutions translate directly into symmetries of the angular frequencies. Thus, the presence of symmetries of solutions facilitates the emergence of weak chimeras and, in fact, most weak chimeras that have been constructed explicitly (Ashwin and Burylko 2015; Panaggio et al. 2016; Bick and Ashwin 2016) are solutions with (instantaneous) symmetries. Is it possible to construct weak chimeras without instantaneous or setwise symmetries for which the angular frequencies have symmetries that are not a property of the solution itself? This question motivates the second contribution. Extending recent persistence results (Bick and Ashwin 2016) that rely on constructing generalized coupling functions between oscillators, we prove a persistence result for weak chimeras with trivial symmetry in weakly coupled populations of phase oscillators. Moreover, we present an explicit example of a $C^\infty$ coupling function that gives rise to a chaotic weak chimera without instantaneous or setwise symmetries in a nontrivially coupled system.

This paper is organized as follows. In Sect. 2, we review some terminology on equivariant dynamics that is needed in the subsequent sections. In Sect. 3, we then apply these notions to general oscillator systems with symmetry which yields a new definition of a weak chimera in terms of symmetries of the angular frequency vector. As we show in Sect. 4, this definition is compatible with previous definitions. In Sect. 5, we prove a persistence result for weak chimeras without instantaneous or average symmetries. Finally, we present an explicit example of a coupling function which gives rise to chaotic weak chimeras with trivial symmetries in Sect. 6 and finish with some concluding remarks.

## Preliminaries

### Quasi-Regular Points

Let ${\mathbf {X}}$ be a compact differentiable manifold with a flow $\Phi _t:{\mathbf {X}}\rightarrow {\mathbf {X}}$, $t\in {\mathbb {R}}$. A point $x\in {\mathbf {X}}$ is *quasi-regular* if the limit

$$\begin{aligned} \lim _{T\rightarrow \infty }\frac{1}{T}\int _{0}^{{T}}f\left( \Phi _t(x)\right) {\mathrm {d}}t \end{aligned}$$

exists for all continuous functions $f:{\mathbf {X}}\rightarrow {\mathbb {R}}$.

#### Theorem 1

(Schwartzman 1957; Oxtoby 1952) The set of points which are not quasi-regular has zero measure with respect to every finite measure on ${\mathbf {X}}$ that is invariant under the flow $\Phi _t$.

### Equivariant Dynamical Systems

Let $F:{\mathbf {X}}\rightarrow {\text {T}}\mathbf{X }$ be a smooth vector field on ${\mathbf {X}}$ where ${\text {T}}\mathbf{X }$ denotes the tangent bundle. Suppose that a group $\Gamma$ acts on ${\mathbf {X}}$. The vector field *F* is $\Gamma$-equivariant if

3 $$\begin{aligned} F(\gamma x) = \hat{\gamma }F(x) \end{aligned}$$

for all $\gamma \in \Gamma$ where $\hat{\gamma }$ is the induced action on the tangent space. A $\Gamma$-equivariant vector field defines a $\Gamma$-*equivariant dynamical system*

4 $$\begin{aligned} \dot{x} = F(x) \end{aligned}$$

on ${\mathbf {X}}$ (Golubitsky and Stewart 2002; Field 2007). For a set $A\subset {\mathbf {X}}$ define the set of *instantaneous symmetries*

5 $$\begin{aligned} T(A)=\left\{ \, \gamma \in \Gamma \,\left| \;\gamma x = x \quad \text { for all }x\in A\right. \right\} \end{aligned}$$

and the set of *symmetries on average* (or setwise symmetries)

6 $$\begin{aligned} \Sigma (A)=\left\{ \, \gamma \in \Gamma \,\left| \;\gamma A = A\right. \right\} . \end{aligned}$$

Clearly, $T(A)\subset \Sigma (A)$. If $\Gamma _x = \left\{ \, \gamma \in \Gamma \,\left| \;\gamma x = x\right. \right\}$ denotes the *stabilizer* or *isotropy subgroup* of $x\in {\mathbf {X}}$ we have $T(A)=\bigcap _{x\in A}\Gamma _x$.

Note that if $\gamma A\cap A=\emptyset$ for all $\gamma \in \Gamma \smallsetminus \left\{ {{\mathrm{id}}}\right\}$, then $\Sigma (A)=\left\{ {{\mathrm{id}}}\right\}$. The converse holds only under additional assumptions (Ashwin 1995). Henceforth, let $\Phi _t:{\mathbf {X}}\rightarrow {\mathbf {X}}$, $t\in {\mathbb {R}}$, denote the flow defined by the differential Eq. (4). A set $A\subset {\mathbf {X}}$ is (forward) *flow-invariant* or *dynamically invariant* if $\Phi _t(A)\subset A$ for all $t\ge 0$. Moreover, *A* is *stable * if for every neighborhood *U* of *A*, there exists an open neighborhood $V\subset U$ of *A* such that $\Phi _t(V)\subset U$ for all $t\ge 0$. A compact stable set *A* is an *attractor* if $A=\omega (x)$ is the $\omega$-limit set of some point $x\in {\mathbf {X}}$.

For attractors and the action of the orthogonal group $O(n)$ on ${\mathbb {R}}^n$, there is the following dichotomy (Melbourne et al. 1993) that characterizes the symmetries on average.

#### Proposition 1

Let $\Gamma \subset O(n)$ be a finite subgroup. For an attractor $A\subset {\mathbb {R}}^n$, we have for any $\gamma \in \Gamma$ either $\gamma A = A$ or $\gamma A\cap A=\emptyset$.

#### Remark 1

The same statement holds for repellers—dynamically invariant sets that are attractors when time is reversed. However, it does not necessarily hold for dynamically invariant sets of saddle type, sets that are attracting (or repelling) in a more general sense, or heteroclinic attractors.

For $\delta >0$, let $B_\delta (A)$ denote an (open) $\delta$-neighborhood of *A*.

#### Corollary 1

Let $\Gamma \subset O(n)$ be a finite subgroup and let $A\subset {\mathbb {R}}^n$ be compact attractor for the flow defined by (4). If $\Sigma (A) = \left\{ {{\mathrm{id}}}\right\}$, then there exists a $\delta >0$ such that $\Sigma (D)=\left\{ {{\mathrm{id}}}\right\}$ for any $D\subset B_\delta (A)$.

#### Proof

Suppose that $\Sigma (A) = \left\{ {{\mathrm{id}}}\right\}$. By Proposition 1, we have $\gamma A \cap A=\emptyset$ for any $\gamma \ne {{\mathrm{id}}}$. Since *A* is closed, there exists a $\delta >0$ such that $\gamma B_\delta (A) \cap B_\delta (A)=\emptyset$. Therefore, $\Sigma (D)=\left\{ {{\mathrm{id}}}\right\}$ for any $D\subset B_\delta (A)$. $\square$

### Equivariant Observables

Suppose that $\Gamma$ acts on both ${\mathbf {X}}$ and ${\mathbb {R}}^m$ for some $m\in {\mathbb {N}}\smallsetminus \left\{ 0\right\}$.

#### Definition 1

A continuous $\Gamma$-equivariant map ${\mathcal {O}}:{\mathbf {X}}\rightarrow {\mathbb {R}}^m$ is an *observable*.

Given a solution *x*(*t*) of (4) with initial condition $x(0)=x^0$, the limit

7 $$\begin{aligned} K_{\mathcal {O}}\left( x^0\right) = \lim _{T\rightarrow \infty }\frac{1}{T}\int _{0}^{{T}}{\mathcal {O}}\left( x(t)\right) {\mathrm {d}}t \end{aligned}$$

(if it exists) is an *average of* ${\mathcal {O}}$ *along the trajectory* *x* (integrate componentwise if $m>1$). The limit exists in particular for every quasi-regular initial condition $x^0$, and henceforth, we will always assume that $x^0\in {\mathbf {X}}$ is quasi-regular when averages (7) are evaluated.

Suppose that $A\subset {\mathbf {X}}$ is dynamically invariant and supports a $\Phi _t$-invariant ergodic probability measure $\mu$. Write

8 $$\begin{aligned} K^\mu _{\mathcal {O}}(A) = \int _A{\mathcal {O}}(x){\mathrm {d}}\mu . \end{aligned}$$

By the Birkhoff ergodic theorem (Katok and Hasselblatt 1995, Theorem 4.1.2), we have

9 $$\begin{aligned} K_{\mathcal {O}}\left( x^0\right) = K^\mu _{\mathcal {O}}(A). \end{aligned}$$

for $\mu$-almost every $x^0\in A$. In particular, the limit (7) exists for $\mu$-almost every $x^0\in A$. For ease of notation, we will simply write $K_{\mathcal {O}}(A)=K^\mu _{\mathcal {O}}(A)$ unless the choice of measure is important. Of course, not every ergodic invariant measure is “physically relevant” since $\mu$ may be singular with respect to the Lebesgue measure. If an attractor *A* supports a Sinai–Ruelle–Bowen (SRB) measure $\mu$ (Young 2002; Katok and Hasselblatt 1995), there is a neighborhood *W* of *A* such that (9) holds for Lebesgue-almost every $x^0\in W$. Thus, the average (8) is observed for “typical” initial conditions with respect to the Lebesgue measure.

Now, $K_{\mathcal {O}}(A)$ has an isotropy group $\Gamma _{K_{\mathcal {O}}(A)}$ and a simple calculation (Golubitsky and Stewart 2002) shows that

10 $$\begin{aligned} \Sigma (A)\subset \Gamma _{K_{\mathcal {O}}(A)}, \end{aligned}$$

that is, any symmetry on average is contained in the isotropy group of the observation.

The converse does not hold for general observables. *Detectives* (Golubitsky and Stewart 2002; Barany et al. 1993; Dellnitz et al. 1994; Ashwin and Nicol 1997) are an important class of observables for which the isotropy is generically equal to the symmetries on average. Given a suitably large $m\in {\mathbb {N}}\smallsetminus \left\{ 0\right\}$, an observable is an (ergodic) detective if for any $\omega$-limit set *A*, there exists an open dense set of near-identity $\Gamma$-equivariant diffeomorphisms $\psi :{\mathbb {R}}^m\rightarrow {\mathbb {R}}^m$ such that $\Gamma _{K_{\mathcal {O}}(\psi (A))}=\Sigma (A)$. Hence, detectives are particular observables to “detect” the symmetries of attractors.

## Weak Chimeras and Symmetries on Average

### Isotropy of Angular Frequencies and Weak Chimeras

The symmetry point of view now allows to define weak chimeras in terms of their symmetries as solutions relative to the symmetries of the system itself. Write $i=\sqrt{-1}$. Let $\Gamma \subset {\mathbf {S}}_n$ be a subgroup that acts transitively on ${\mathbb {C}}_\bullet ^n:= ({\mathbb {C}}\smallsetminus \left\{ 0\right\} )^n$ by permuting coordinates and suppose that $F:{\mathbb {C}}_\bullet ^n\rightarrow {\mathbb {C}}^n$ is $\Gamma$-equivariant. The map $F=(F_1, \cdots , F_n)$ determines a dynamical system on ${\mathbb {C}}_\bullet ^n$ where the evolution of $z = (z_1, \cdots , z_k)$ 1 is given by

11 $$\begin{aligned} \dot{z}_k = z_k F_k(z). \end{aligned}$$

For $$k\in \left\{ 1, \cdots , n\right\} $$ define

12 $$\begin{aligned} C_k(z) = \frac{z_k}{\left| z_k\right| }. \end{aligned}$$

In the following, we assume that *F* is such that (a) the dynamics of (11) are well defined on ${\mathbb {C}}_\bullet ^n$, (b) we have $\omega (z)\subset {\mathbb {C}}_\bullet ^n$ for all $z\in {\mathbb {C}}_\bullet ^n$ and (c) the derivative $C_k^{\prime }(z(t)) := \frac{{\mathrm {d}}}{{\mathrm {d}}t}C_k(z(t))$ exists for any trajectory *z*(*t*). These assumptions are easy to work with but can be relaxed as one typically only needs well-defined dynamics on a neighborhood of ${\mathbf {T}}^n\subset {\mathbb {C}}_\bullet ^n$.

Note that $C_k(z_1, \cdots , z_n)$ projects onto the unit circle in the *k*th coordinate. Let $\gamma _T$ denote the parametrized curve in ${\mathbb {C}}_\bullet ^n$ determined by a solution *z*(*t*) of (11) for $t\in [0, T]$. The change in argument of $z_k$ along $\gamma _T$ is given by

13 $$\begin{aligned} \Delta \arg C_k(\gamma _T) = \frac{1}{i}\int _0^{{T}} \frac{C_k^{\prime }(z(t))}{C_k(z(t))}{\mathrm {d}}t = \int _0^{{T}} {{\mathrm{Im}}}(F_k(z(t))){\mathrm {d}}t. \end{aligned}$$

Thus, we obtain the *average angular frequency in the kth coordinate* (equivalent to the average winding number when multiplied by $2\pi$)

14 $$\begin{aligned} \Omega _k\left( z^0\right) := \lim _{T\rightarrow \infty }\frac{1}{T}\int _0^{{T}} {{\mathrm{Im}}}\left( F_k(z(t))\right) {\mathrm {d}}t \end{aligned}$$

along a trajectory *z*(*t*) with initial condition $z^0$.

#### Definition 2

The vector

$$\begin{aligned} \Omega \left( z^0\right) = \left( \Omega _1\left( z^0\right) , \cdots , \Omega _n\left( z^0\right) \right) \end{aligned}$$

is the *angular frequency vector* of the trajectory with initial condition $z^0\in {\mathbb {C}}_\bullet ^n$.

Since ${\mathbf {S}}_n$ also acts on ${\mathbb {R}}^n$ by permuting indices, ${{\mathrm{Im}}}(F):{\mathbb {C}}_\bullet ^n\rightarrow {\mathbb {R}}^n$ is a $\Gamma$-equivariant observable for (11) and

$$\begin{aligned} \Omega \left( z^0\right) = K_{{{\mathrm{Im}}}(F)}\left( z^0\right) , \end{aligned}$$

that is, the angular frequency vector is the observation of ${{\mathrm{Im}}}(F)$ along a trajectory. For a compact and invariant set $A\subset {\mathbb {C}}_\bullet ^n$ with an unique ergodic invariant measure, we write $\Omega (A) = K_{{{\mathrm{Im}}}(F)}(A)$ for the *angular frequency vector of* *A*.

The symmetries of the system (11) now allow to phrase angular frequency synchronization in terms of the isotropy of the angular frequency vector. An observation of ${{\mathrm{Im}}}(F)$ has isotropy subgroup $\Gamma _{\Omega (A)}\subset \Gamma$. This motivates a definition of a weak chimera (Ashwin and Burylko 2015)—originally limited to networks of phase oscillator—to more general oscillator systems (11).

#### Definition 3

A compact, connected, chain-recurrent and dynamically invariant set $A\subset {\mathbb {C}}_\bullet ^n$ is a *weak chimera* for (11) if

$$\begin{aligned} \left\{ {{\mathrm{id}}}\right\} \subsetneq \Gamma _{\Omega \left( \varphi ^0\right) } \subsetneq \Gamma \end{aligned}$$

for all $\varphi ^0\in A$. If a weak chimera *A* supports an SRB measure, then it is called *observable* and we have

$$\begin{aligned} \left\{ {{\mathrm{id}}}\right\} \subsetneq \Gamma _{\Omega (A)} \subsetneq \Gamma . \end{aligned}$$

#### Remark 2

Asymptotic winding (or rotation) numbers can be defined in a more general setting: they quantify how trajectories of a given flow wind around a topological space ${\mathbf {X}}$; cf. Schwartzman (1957), Walsh (1995) for details. These winding numbers are defined for continuous maps $f:{\mathbf {X}}\rightarrow S^1=\left\{ \, z\in {\mathbb {C}}\,\left| \;\left| z\right| =1\right. \right\}$. For spaces with finitely generated homology, it suffices to evaluate winding numbers for maps $f_k$ corresponding to a basis of the first cohomology (Schwartzman 1957).

Here, we have ${\mathbf {X}}={\mathbb {C}}_\bullet ^n$ and the maps $C_k$ defined above correspond to the generators of the homology of ${\mathbb {C}}_\bullet ^n$. Since we consider flows given by a $\Gamma$-equivariant differential equation, we characterize weak chimeras by the symmetry properties of the asymptotic winding numbers. In the language of asymptotic cycles, these are solutions where for certain “directions,” the winding behavior is the same, while for other directions, it is distinct. This suggests that the notion can be further extended to equivariant dynamical systems on more general ${\mathbf {X}}$ with nontrivial homology.

Note that the weak chimeras of Definition 3 are defined solely in terms of the symmetry properties of the system. Moreover, the definition extends beyond the weak coupling limit of interacting limit cycle oscillators (Ashwin and Swift 1992): Systems of the form (11) describe dynamical systems close to a Hopf bifurcation (Ashwin and Rodrigues 2016) or more general oscillator models where “amplitude-mediated chimeras” have been observed (Sethia et al. 2013). Moreover, the next proposition asserts that the change in argument of $z_k$ along $C_\ell (\gamma _T)$ cannot be bounded to obtain nontrivial winding numbers; such dynamics are observed for “pure amplitude chimeras” (Zakharova et al. 2014), and thus, our definition is sufficiently general to provide a rigorous framework for such chimeras.

#### Proposition 2

Suppose that *z*(*t*) is a solution of (11) such that there are $j\ne \ell$, $M,R>0$ such that $\Delta \arg C_\ell (\gamma _T)<M$ and $\Delta \arg C_j(\gamma _T)>RT$ for all *T*. Then, $\Gamma _{\Omega (A)}\subsetneq \Gamma$.

#### Proof

Immediate from $$\Omega _k\left( z^0\right) = \lim _{T\rightarrow \infty }\frac{1}{T}\Delta \arg C_k(\gamma _T)$$. $$\square $$

Definition 3 is compatible with the action of the symmetry group on ${\mathbb {C}}_\bullet ^n$.

#### Proposition 3

If $A\subset {\mathbf {T}}^n$ is a weak chimera, so is $\gamma A$ for any $\gamma \in \Gamma$.

#### Proof

The assertion follows directly from $\Gamma$-equivariance of *F*. $\square$

This implies in particular that the isotropy of the angular frequency vectors are conjugate if weak chimeras are related by symmetry. If $\gamma \in \Sigma (A)$, then $\Omega (\gamma A)=\Omega (A)$, that is, the angular frequency vectors (and therefore the isotropy) are identical.

### Symmetries Imply Frequency Synchronization

Intuitively speaking, a weak chimera *A* consists of solutions of (11) along which the average angular frequencies have some symmetries but not too many. Inclusion (10) implies

15 $$\begin{aligned} \left\{ {{\mathrm{id}}}\right\} \subset T(A)\subset \Sigma (A)\subset \Gamma _{\Omega (A)}\subset \Gamma \subset {\mathbf {S}}_n. \end{aligned}$$

Consequently, if a solution has nontrivial instantaneous symmetry, then the corresponding angular frequency vector has nontrivial isotropy. Similarly, the angular frequency vector of dynamically invariant sets with nontrivial setwise symmetry has nontrivial isotropy. For invariant sets with nontrivial (setwise or instantaneous) symmetry, (15) implies that one condition of Definition 3 is automatically satisfied. In that sense, the presence of symmetries “facilitates” the occurrence of weak chimera states.

More generally speaking, symmetries of the system give sufficient conditions for angular frequency synchronization (Golubitsky et al. 2006). These are not necessary as there may be other dynamically invariant subspaces where oscillators are phase and frequency locked which are not induced by symmetry but rather by balanced polydiagonals of colored graphs (Antoneli and Stewart 2006).

## Weak Chimeras for Networks of Phase Oscillators

Definition 3 relates to the original definition of a weak chimera for networks of coupled phase oscillators (Ashwin and Burylko 2015). We will not restrict ourselves to systems (1) but consider a more general setup that may include, for example, nonpairwise interactions (Ashwin and Rodrigues 2016; Bick et al. 2016). More precisely, let ${\mathbf {X}}={\mathbf {T}}^n$ and let $\Gamma \subset {\mathbf {S}}_n$ act transitively on ${\mathbf {T}}^n$ by permuting indices. A smooth $\Gamma$-equivariant vector field $Y:{\mathbf {T}}^n\rightarrow {\mathbb {R}}^n$ now defines a $\Gamma$-equivariant dynamical system

16 $$\begin{aligned} \dot{\varphi }= Y(\varphi ). \end{aligned}$$

that describes the evolution of $n$ phase oscillators where the state of oscillator *k* is given by $\varphi _k\in {\mathbf {T}}$.

Write $z_k = \exp (i\varphi _k)$ and identify initial conditions $\varphi ^0\in {\mathbf {T}}^n$ with $z^0\in {\mathbb {C}}_\bullet ^n$. The dynamics of (16) can be embedded in ${\mathbb {C}}_\bullet ^n$ as

17 $$\begin{aligned} \dot{z}_k = z_k\left( iY_k(\varphi )\right) . \end{aligned}$$

Therefore,

18 $$\begin{aligned} \Omega _k\left( \varphi ^0\right) := K_{Y_k}\left( z^0\right) = \lim _{T\rightarrow \infty }\frac{1}{T}\int _0^{{T}} Y_k(\varphi (t)){\mathrm {d}}t \end{aligned}$$

and if $A\subset {\mathbf {T}}^n$ is compact, dynamically invariant supporting an SRB measure then

$$\begin{aligned} \Omega \left( \varphi ^0\right) = K_Y(A) \end{aligned}$$

is the *angular frequency vector* for *A*. Moreover, with (16), we have

19 $$\begin{aligned} \int _{0}^{{T}}Y_k(\varphi (t)){\mathrm {d}}t = \int _{0}^{{T}}\dot{\varphi }_k(t){\mathrm {d}}t = \hat{\varphi }_k(T)-\hat{\varphi }_k(0) \end{aligned}$$

where $\hat{\varphi }$ is a continuous lift of the trajectory $\varphi (t)$ to ${\mathbb {R}}^n$. Thus,

$$\begin{aligned} \Omega _k\left( \varphi ^0\right) = \lim _{T\rightarrow \infty }\frac{\hat{\varphi }(T)}{T} \end{aligned}$$

as given by (2). Note also that $\Omega _k(A)$ correspond to the average frequency defined in (Golubitsky et al. 2006) and relates to the rotation vector for torus maps (Misiurewicz and Ziemian 1989).

Compared to the original definition of a weak chimera in (Ashwin and Burylko 2015), Definition 3 is more restrictive. More precisely, for *A*, we require that frequency synchronization is only relevant for a weak chimera if the oscillators are related by symmetry. By contrast, the original definition considers the set

20 $$\begin{aligned} \Theta (A) = \left\{ \, \gamma \in {\mathbf {S}}_n\,\left| \;\gamma \Omega (A)=\Omega (A)\right. \right\} \end{aligned}$$

rather than the isotropy $\Gamma _{\Omega _k(A)}$. Note that $\Theta (A)$ may be strictly larger than $\Gamma _{\Omega (A)}$. For example if ${\mathbb {Z}}_n= {\mathbb {Z}}/n{\mathbb {Z}}\subset {\mathbf {S}}_n$ denotes the cyclic group and *X* is ${\mathbb {Z}}_n$ equivariant but not ${\mathbf {S}}_n$-equivariant and $\varphi ^0_1=\cdots =\varphi ^0_n$ [for example, a nonlocally coupled ring of phase oscillators (Kuramoto and Battogtokh 2002)] is a solution of $\dot{\varphi }= X(\varphi )$ then $\Theta \big (\left\{ \varphi ^0\right\} \big ) = {\mathbf {S}}_n\supsetneq {\mathbb {Z}}_n$.

## Persistence of Weak Chimeras Without Symmetry on Average for Diffusively Coupled Phase Oscillators

The inclusions (15) in Sect. 3.2 imply that any (nontrivial) instantaneous or average symmetry of a dynamically invariant set gives nontrivial isotropy of the angular frequency vector. This is the case for the weak chimeras constructed in (Ashwin and Burylko 2015; Panaggio et al. 2016; Bick and Ashwin 2016). In this section, we construct weak chimeras with trivial average symmetries for systems consisting of two weakly interacting populations of phase oscillators.

### Coupling Function Separability for Symmetric Diffusively Coupled Phase Oscillators

For $\phi , \psi \in {\mathbf {T}}^n$ define $X=(X_1, \cdots , X_n)$ by

21 $$\begin{aligned} X_k(\phi , \psi ) := \frac{1}{n}\sum _{j=1}^{n}g\left( \phi _k-\psi _j\right) . \end{aligned}$$

The dynamics of a fully symmetric network of $n$ phase oscillators with coupling function *g* is given by the ${\mathbf {S}}_n$-equivariant dynamical system on ${\mathbf {T}}^n$ where

22 $$\begin{aligned} \dot{\varphi }_k = Y_k(\varphi ) := X_k(\varphi , \varphi ) \end{aligned}$$

describes the evolution of the *k*th oscillator.^2^ We may assume $g(0)=0$ by going to suitable co-rotating reference frame, $\varphi _k \mapsto \varphi _k - {\omega }t$. If the choice of coupling function *g* is important, we write $Y^{(g)}$ or $X^{(g)}$ to highlight the dependency. Reducing the continuous ${\mathbf {T}}$ symmetry of (22) allows to set $\varphi _1=0$. Because of the ${\mathbf {S}}_n$-equivariance, the *canonical invariant region*

23 $$\begin{aligned} {\mathcal {C}}:=\left\{ \, \varphi \in {\mathbf {T}}^n\,\left| \;0=\varphi _1<\cdots<\varphi _n< 2\pi \right. \right\} \end{aligned}$$

is dynamically invariant. It is bounded by hypersurfaces corresponding to cluster states with $\varphi _k=\varphi _{k+1}$, and there is a residual ${\mathbb {Z}}_n$ symmetry on ${\mathcal {C}}$ (Ashwin and Swift 1992; Ashwin et al. 2016a).

For a compact flow-invariant set $A\subset {\mathbf {T}}^n$ define

24 $$\begin{aligned} \Xi (A) = \bigcup _{k\ne j}\left\{ \, \varphi _k-\varphi _j\,\left| \;\varphi \in A\right. \right\} . \end{aligned}$$

Note that $\Xi (\gamma A)=\Xi (A)$ for all $\gamma \in {\mathbf {S}}_n$.

#### Definition 4

Two sets $A_1, A_2\subset {\mathcal {C}}$ are *coupling function separated* if there are open intervals $Q_{A_1}, Q_{A_2}\subset {\mathbf {T}}$ with $\Xi (A_\ell )\subset Q_{A_\ell }$, $\ell =1,2$, and

$$\begin{aligned} \overline{Q}_{A_1}\cap \overline{Q}_{A_2}=\emptyset \end{aligned}$$

where the bar denotes topological closure.

For $Q\subset {\mathbf {T}}$ define $\Xi ^{-1}(Q):=\left\{ \, \varphi \in {\mathbf {T}}\,\left| \;\Xi (\left\{ \varphi \right\} )\subset Q\right. \right\}$ and

$$\begin{aligned} W^{(g)}(Q) := \Big [\min _{k\in \left\{ 1, \cdots , n\right\} }\inf _{\varphi \in \Xi ^{-1}(Q)} Y_k^{(g)}(\varphi ), \max _{k\in \left\{ 1, \cdots , n\right\} }\sup _{\varphi \in \Xi ^{-1}(Q)} Y_k^{(g)}(\varphi )\Big ]. \end{aligned}$$

#### Lemma 1

1. If $A\subset {\mathcal {C}}$ with $\Xi (A)\subset Q$ is dynamically invariant for the dynamics of (22) with coupling function *g*, then $\Omega _k\left( \varphi ^0\right) \in W^{(g)}(Q)$.
2. Suppose that $A_\ell \subset {\mathcal {C}}$, $\ell =1,2$, are compact and coupling function separated with separating sets $Q_{A_\ell }$. Then for any $\eta \ge 0$, we can find a coupling function $\hat{g}$ such that
  $$\begin{aligned} B_\eta \left( W^{(\hat{g})}\left( Q_{A_1}\right) \right) \cap B_\eta \left( W^{(\hat{g})}\left( Q_{A_2}\right) \right) =\emptyset . \end{aligned}$$
3. Let $A_\ell$ be as above and let $A_1^{\prime },A_2^{\prime }\subset {\mathcal {C}}$ be dynamically invariant for the dynamics of (22) with $\Xi (A_\ell ^{\prime })\subset Q_{A_\ell }$. Then, there is a coupling function $\hat{g}$ such that $A_1^{\prime },A_2^{\prime }$ are dynamically invariant for the dynamics of (22) with $\hat{g}$ and
  $$\begin{aligned} \Omega _k\left( \varphi ^0_{A_1^{\prime }}\right) \ne \Omega _j\left( \varphi ^0_{A_2^{\prime }}\right) \end{aligned}$$
  for all *k*, *j* and $\varphi ^0_{A_\ell ^{\prime }}\in A_\ell ^{\prime }$.

#### Proof

To prove (1), note first that invariance of ${\mathcal {C}}$ implies that $\Omega _j\left( \varphi ^0\right) =\Omega _k\left( \varphi ^0\right)$ for all *k*, *j* and $\varphi ^0\in A$. Standard integral estimate for (18) yield

$$\begin{aligned} \Omega _k\left( \varphi ^0\right) \in \Big [\inf _{\varphi \in A} Y_k^{(g)}(\varphi ), \sup _{\varphi \in A} Y_k^{(g)}(\varphi )\Big ] \subset W^{(g)}(Q). \end{aligned}$$

To prove (2), consider coupling functions $\hat{g}$ with $\hat{g}(\phi )=g(\phi )+a_\ell$ for all $\phi \in Q_{A_\ell }$, $\ell =1,2$. Since

$$\begin{aligned} Y_k^{(\hat{g})}(\varphi ) = \frac{1}{n}\sum _{j=1}^{n}\left( g(\phi _k-\psi _j)+a_\ell \right) = a_\ell + Y_k^{(g)}(\varphi ) \end{aligned}$$

for all $\varphi$ with $\Xi (\left\{ \varphi \right\} )\subset Q_{A_\ell }$ we have

$$\begin{aligned} W^{(\hat{g})}(Q_{A_\ell }) = \left[ \inf W^{(g)}(Q_{A_\ell }) + a_\ell , \sup W^{(g)}(Q_{A_\ell }) + a_\ell \right] . \end{aligned}$$

For a given $\eta \ge 0$, choose $a_1, a_2$ such that

$$\begin{aligned} B_\eta \left( W^{(g)}(Q_{A_1})\right) \cap B_\eta \left( W^{(g)}(Q_{A_2})\right) =\emptyset \end{aligned}$$

to obtain the desired coupling function $\hat{g}$.

Note that replacing *g* by $\hat{g}$ as above preserves dynamically invariant sets *A* with $\Xi (A)\subset Q_{A_\ell }$. Claim (3) now follows from (1) and (2) with $\eta =0$. $\square$

#### Remark 3

The notion of function coupling separability and Lemma 1 generalize to a finite number of sets $A_1, \cdots , A_r$. The function $g-\hat{g}$ can typically be chosen to be $C^\infty$.

### Relative Equilibria with Trivial Symmetry

We now show that choosing the coupling function appropriately in an arbitrarily small neighborhood of zero gives rise to asymptotically stable relative equilibria with trivial symmetry for (22).

Let $0=\alpha _1<\cdots<\alpha _n<2\pi$. The function

25 $$\begin{aligned} \varphi ^\star (t)=\left( \alpha _1+t{\omega ^\star }, \cdots , \alpha _n+t{\omega ^\star }\right) \in {\mathcal {C}}\end{aligned}$$

with $\omega ^\star = \frac{1}{n}\sum _{j=2}^ng(-\alpha _j)$ is a relative equilibrium of (22) for any coupling function *g* such that

26 $$\begin{aligned} \frac{1}{n}\sum _{j\ne k}\left( g(\alpha _k-\alpha _j)-g(-\alpha _j)\right) = 0 \end{aligned}$$

for all $k=2, \cdots , n$. For a relative equilibrium, we have

27 $$\begin{aligned} \Xi \left( \left\{ \varphi ^\star \right\} \right) =\bigcup _{k\ne j}\left\{ \alpha _k-\alpha _j\right\} \subset \left[ -2\alpha _{n}, 2\alpha _{n}\right] \subset {\mathbf {T}}. \end{aligned}$$

In particular, we have a relative equilibrium if the coupling function *g* vanishes on $\Xi (\left\{ \varphi ^\star \right\} )$. Since $\alpha _n$ can be chosen arbitrarily small, the relative equilibrium can be chosen arbitrarily close to the fully synchronized solution $\varphi _1=\cdots =\varphi _n$. We have $\Omega (\left\{ \varphi ^\star \right\} ) = (\omega ^\star , \cdots , \omega ^\star )$.

Stability of the relative equilibrium is determined by the linearization

28 $$\begin{aligned} \frac{\partial Y_k}{\partial \varphi _j} = {\left\{ \begin{array}{ll} \frac{1}{n}\sum _{l\ne k}g^{\prime }(\alpha _k-\alpha _l) &{} \quad \text {if }\,k=j,\\ -\frac{1}{n}g^{\prime }(\alpha _k-\alpha _j) &{} \quad \text {otherwise}. \end{array}\right. } \end{aligned}$$

By choosing the coupling function appropriately on $\Xi (A)$, the relative equilibrium will be asymptotically stable. For example, if $g^{\prime }(\phi )=0$ for $\phi <0$ and $g^{\prime }(\phi )<0$ for $\phi >0$, we have a lower triangular matrix with negative values on the diagonal (apart from one zero eigenvalue) implying that $\varphi ^\star$ is asymptotically stable.

#### Lemma 2

Let $\varphi ^\star (t)$ be a relative equilibrium of (22) as defined in (25). If $\left| \alpha _n\right| <\frac{\pi }{2}$ then $T(\left\{ \varphi ^\star \right\} )=\Sigma (\left\{ \varphi ^\star \right\} )=\left\{ {{\mathrm{id}}}\right\}$.

#### Proof

It suffices to show that $\Sigma (\left\{ \varphi ^\star \right\} )=\left\{ {{\mathrm{id}}}\right\}$. Assume that $\gamma \in \Sigma (\left\{ \varphi ^\star \right\} )$ with $\gamma \ne {{\mathrm{id}}}$. Then, there exists a $\tau \ge 0$ such that

$$\begin{aligned} \alpha _{\gamma k} = \alpha _{k}+\tau \omega ^\star \mod 2\pi \end{aligned}$$

for all *k*. Recall that $0=\alpha _1<\cdots<\alpha _n<2\pi$. Since $\gamma \ne {{\mathrm{id}}}$, $\gamma$ permutes some indices. Assume without loss of generality that $\alpha _{\gamma 1}>\alpha _1$ and $\alpha _{\gamma 2}<\alpha _2$. We have $\alpha _{\gamma 1}-\alpha _1 = \alpha _{\gamma 2}-\alpha _2 = \tau \omega ^\star \mod 2\pi$. But since $\alpha _{\gamma 1}-\alpha _1>0$ and $\alpha _{\gamma 2}-\alpha _2<0$, there has to be an $m>0$ such that $\alpha _{\gamma 1}-\alpha _1 -\alpha _{\gamma 2}+\alpha _2 = 2m\pi$. This is a contradiction since $\left| \alpha _{\gamma 1}-\alpha _1 - \alpha _{\gamma 2}+\alpha _2\right| \le 4\alpha _n<2\pi$. $\square$

### Weak Chimeras with $\Sigma (A)=\left\{ {{\mathrm{id}}}\right\}$ in Weakly Coupled Populations of Phase Oscillators

Chaotic weak chimeras have many features associated with classical chimera states including positive maximal Lyapunov exponents. Hence, rather than using a hyperbolicity argument to construct nonchaotic weak chimeras as in (Ashwin and Burylko 2015), we aim to construct weak chimeras with $\Sigma (A)=\left\{ {{\mathrm{id}}}\right\}$ in a more general setup which allows for positive maximal Lyapunov exponents. To this end, we extend recent results from (Bick and Ashwin 2016) with respect to the instantaneous and setwise symmetries of the constructed sets.

Coupling two populations of $n$ oscillators, whose uncoupled dynamics are given by (22), defines a dynamical system on ${\mathbf {T}}^{2n}$. More explicitly, write $\varphi = (\varphi _{1}, \varphi _{2})\in {\mathbf {T}}^n\times {\mathbf {T}}^n={\mathbf {T}}^{2n}$, $\varphi _{\ell }=(\varphi _{\ell ,1}, \cdots , \varphi _{\ell ,n})$ and consider the product system

29 $$\begin{aligned} \begin{aligned} \dot{\varphi }_{1}&= {\mathcal {Y}}_1^{(g,\varepsilon )}(\varphi _{1}, \varphi _2) = Y^{(g)}(\varphi _{1}) + \varepsilon X^{(g)}(\varphi _{1},\varphi _{2}),\\ \dot{\varphi }_{2}&= {\mathcal {Y}}_2^{(g,\varepsilon )}(\varphi _{1}, \varphi _2) = Y^{(g)}(\varphi _{2}) + \varepsilon X^{(g)}(\varphi _{2},\varphi _{1}), \end{aligned} \end{aligned}$$

with $Y^{(g)}, X^{(g)}$ as in (22), (21). Observe that for $\varepsilon =0$, the system decouples into two identical groups of *n* oscillators—both of which with nontrivial dynamics (22). For $\varphi ^0\in {\mathbf {T}}^{2n}$, we denote the asymptotic angular frequency of the oscillator with phase $\varphi _{\ell ,k}$ by $\Omega _{\ell ,k}\left( \varphi ^0\right) =\Omega _{\ell ,k}^{(g,\varepsilon )}\left( \varphi ^0\right)$.

Let $\Gamma = {\mathbf {S}}_n\wr {\mathbf {S}}_{2}$ where $\wr$ is the wreath product. The system (29) is $\Gamma$-equivariant (Dionne et al. 1996); we have $\Gamma = {\mathbf {S}}_n\wr {\mathbf {S}}_{2}=({\mathbf {S}}_n)^2\rtimes {\mathbf {S}}_{2}$ where the elements of ${\mathbf {S}}_n$ permute the oscillators within each group of *n* oscillators and the action of ${\mathbf {S}}_{2}$ permutes the two groups. Observe that this is only a semidirect product $\rtimes$ as the two sets of permutations do not necessarily commute. The oscillators are indistinguishable as this group acts transitively on the oscillators.

Weak chimeras in the product system persist for weak coupling $0\le \varepsilon \ll 1$. As in (Bick and Ashwin 2016), we call a dynamically invariant set *A* is *sufficiently stable* if there is an open neighborhood of *A* on which a Lyapunov function is defined. The persistence theorem for weak chimeras (Bick and Ashwin 2016, Theorem 4) generalizes to coupling function separated sets that are sufficiently stable.

#### Theorem 2

Suppose that *g* is a coupling function such that $A_1, A_2\subset {\mathcal {C}}$ are compact, forward invariant, coupling function separated and sufficiently stable sets for the dynamics of (22) with $Y^{(g)}$. Then, for any sufficiently small $\delta >0$, there exist a smooth coupling function $\hat{g}$ and $\varepsilon _0>0$ such that for any $0\le \varepsilon <\varepsilon _0$, the weakly coupled product system (29) with *g* replaced by $\hat{g}$ has a sufficiently stable weak chimera $A^{(\varepsilon )}$ with $A^{(\varepsilon )} \subset B_\delta (A_1\times A_2)$.

#### Proof

First, consider the coupling function separated sets $A_1, A_2\subset {\mathbf {T}}^n$ as dynamically invariant sets for (22), a factor of the uncoupled system. Suppose that $Q_{A_1}, Q_{A_2}$ are the separating sets and for a coupling function $\tilde{g}$ define $M(\tilde{g}) := \max _{(\varphi _1, \varphi _2)\in {\mathbf {T}}^{2n}}\big |X^{(\tilde{g})}(\varphi _1, \varphi _2)\big |<\infty$. Now, choose a coupling function $\hat{g}$ according to Lemma 1(2) for $\eta =1$ and fix $\varepsilon _1:=M(\hat{g})^{-1}$. For any $0\le \varepsilon <\varepsilon _1$, we have

30 $$\begin{aligned} B_{\varepsilon M(\hat{g})}\left( W^{(\hat{g})}(Q_{A_1})\right) \cap B_{\varepsilon M(\hat{g})}\left( W^{(\hat{g})}(Q_{A_2})\right) =\emptyset . \end{aligned}$$

since $$\varepsilon M(\hat{g})<\varepsilon _1 M(\hat{g})=1$$.

Now, consider the product system (29). For any sufficiently small $\delta >0$, we obtain $\varepsilon _2>0$ and compact invariant sets $A^{(\varepsilon )}\subset B_\delta (A_1\times A_2)$ for all $0\le \varepsilon <\varepsilon _2$ as in (Bick and Ashwin 2016). Set $\varepsilon _0 < \min \left\{ \varepsilon _1, \varepsilon _2\right\}$. By restricting $\delta$ appropriately, the sets $A^{(\varepsilon )}$ are weak chimeras.

To show that $\Gamma _{\Omega \left( \varphi ^0\right) }\ne \left\{ {{\mathrm{id}}}\right\}$, assume that $\delta$ is so small that $B_\delta (A_1\times A_2)\subset {\mathcal {C}}^2$. This implies that the phase ordering within each population is preserved. Hence, for given $\ell =1,2$, we have

$$\begin{aligned} \Omega _{\ell ,k}^{(\hat{g},\varepsilon )}\left( \varphi ^0\right) = \Omega _{\ell ,j}^{(\hat{g},\varepsilon )}\left( \varphi ^0\right) =: \Omega _{\ell ,*}^{(\hat{g},\varepsilon )}\left( \varphi ^0\right) \end{aligned}$$

for all *k*, *j* and any $\varphi ^0\in A^{\varepsilon }$. Thus, $\Gamma _{\Omega \left( \varphi ^0\right) }\ne \left\{ {{\mathrm{id}}}\right\}$.

It remains to be shown that $\Gamma _{\Omega \left( \varphi ^0\right) }\ne \Gamma$. Let $A_\ell ^{(\varepsilon )}$ denote the projection of $A^{(\varepsilon )}$ onto $\varphi _\ell$. Now, assume that $\delta$ is sufficiently small such that $A^{(\varepsilon )} \subset B_\delta (A_1\times A_2)$ implies that $\Xi \big (A_\ell ^{(\varepsilon )}\big )\subset Q_{A_\ell }$ for all $0\le \varepsilon <\varepsilon _0$. Since ${\mathcal {Y}}_1^{(\hat{g},\varepsilon )}(\varphi _{1}, \varphi _2) = Y^{(\hat{g})}(\varphi _{1}) + \varepsilon X(\varphi _{1},\varphi _{2})$ integral estimates as in Lemma 1(1) imply that

$$\begin{aligned} \Omega _{\ell ,*}^{(\hat{g},\varepsilon )}\in B_{\varepsilon _0M}\left( W^{(\hat{g})}(Q_{A_\ell })\right) \end{aligned}$$

for all $0\le \varepsilon <\varepsilon _0$. By choice of $\hat{g}$, Eq. (30) now implies $\Omega _{1,*}^{(\hat{g},\varepsilon )}\left( \varphi ^0\right) \ne \Omega _{2,*}^{(\hat{g},\varepsilon )}\left( \varphi ^0\right)$ for all $\varphi ^0\in A^{(\varepsilon )}$. Thus, $\Gamma _{\Omega \left( \varphi ^0\right) }\ne \Gamma$ and $A^{(\varepsilon )}$ is a weak chimera. $\square$

The following statement asserts that trivial symmetries in the factors carry over to the product dynamics.

#### Lemma 3

Let $A_1, A_2\subset {\mathbf {T}}^n$ be coupling function separated attractors for (22) with $\Sigma (A_1)=\Sigma (A_2)=\left\{ {{\mathrm{id}}}\right\}$. Then $\Sigma (A_1\times A_2)=\left\{ {{\mathrm{id}}}\right\}$ for the product system (29).

#### Proof

Write ${\mathbf {S}}_{2} = \left\{ {{\mathrm{id}}}, \tau \right\}$. For any $\gamma \in ({\mathbf {S}}_n)^2$, we have $(\gamma , {{\mathrm{id}}})(A_1\times A_2)\cap (A_1\times A_2) = \emptyset$ in ${\mathbf {T}}^{2n}$ by assumption. Since $A_1$ and $A_2$ are coupling function separated, we have $A_1\cap A_2=\emptyset$ in ${\mathbf {T}}^n$. Write $\Gamma V = \bigcup _{\gamma \in \Gamma }\gamma V$, $V\in \left\{ A_1,A_2\right\}$. The fact that $\Xi (\gamma A_1)=\Xi (A_1)$ implies $\Gamma A_1\cap \Gamma A_2=\emptyset$ in ${\mathbf {T}}^n$. Since $(\gamma , \tau )(A_1\times A_2)\subset \Gamma A_2\times \Gamma A_1$ for any $\gamma \in ({\mathbf {S}}_n)^2$, we have $(\gamma , \tau )(A_1\times A_2)\cap (A_1\times A_2)=\emptyset$ and by Proposition 1, the claim follows. $\square$

Combining the perturbation result Theorem 2 with the symmetry considerations, we can now state the main theorem of this section. In order to apply Theorem 2, we make a slightly stronger assumption concerning stability of the relative periodic orbits.

#### Theorem 3

Suppose that *g* is a coupling function such that for the ${\mathbf {S}}_n$-equivariant dynamics of (22) with $Y^{(g)}$, the set $A_{{\text {coh}}}=\left\{ \varphi ^{\star }\right\}$ is a sufficiently stable relative equilibrium and $A_{{\text {inc}}}\subset {\mathbf {T}}^n$ is sufficiently stable attractor that are coupling function separated and $\Sigma (A_{{\text {coh}}})=\Sigma (A_{{\text {inc}}})=\left\{ {{\mathrm{id}}}\right\}$. Then, for any sufficiently small $\delta >0$, there is a coupling function $\hat{g}$ and $\varepsilon _0>0$ such that for any $0\le \varepsilon <\varepsilon _0$, there is a weak chimera $A^{(\varepsilon )}\subset B_\delta (A_{{\text {coh}}}\times A_{{\text {inc}}})$ with $\Sigma (A^{(\varepsilon )})=\left\{ {{\mathrm{id}}}\right\}$ for the ${\mathbf {S}}_n\wr {\mathbf {S}}_{2}$-equivariant dynamics of (29) with $\hat{g}$.

#### Proof

Suppose that $\Sigma (A_{{\text {coh}}})=\Sigma (A_{{\text {inc}}})=\left\{ {{\mathrm{id}}}\right\} \subset {\mathbf {S}}_n$. By Lemma 3, we have $\Sigma (A_{{\text {coh}}}\times A_{{\text {inc}}})=\left\{ {{\mathrm{id}}}\right\} \subset {\mathbf {S}}_n\wr {\mathbf {S}}_{2}$. Since $A_{{\text {coh}}}\times A_{{\text {inc}}}$ is assumed to be an attractor, Corollary 1 implies that there exists a $\delta _0>0$ such that for any invariant set $A\subset B_{\delta _0}(A_{{\text {coh}}}\times A_{{\text {inc}}})$, we have $\Sigma (A)=\left\{ {{\mathrm{id}}}\right\} \subset {\mathbf {S}}_n\wr {\mathbf {S}}_{2}$.

For sufficiently small $0<\delta <\delta _0$, Theorem 2 yields an $\varepsilon _0>0$ and weak chimeras $A^{(\varepsilon )}\subset B_\delta (A_{{\text {coh}}}\times A_{{\text {inc}}})$ for all $0\le \varepsilon <\varepsilon _0$. Since $\delta <\delta _0$, the argument above implies that $\Sigma (A^{(\varepsilon )})=\left\{ {{\mathrm{id}}}\right\}$ for all such $\varepsilon$. $\square$

#### Remark 4

1. In fact, the condition that $A_{{\text {inc}}}$ is a relative equilibrium is not necessary. Theorem 3 holds for any compact, sufficiently stable attractor $A_{{\text {coh}}}\subset {\mathcal {C}}$ that is coupling function separated from $A_{{\text {inc}}}$. Moreover, the same statement holds for (sufficiently unstable) repellers.
2. Even if the weak chimera $A^{(0)}=A_{{\text {coh}}}\times A_{{\text {inc}}}$ is observable, extra assumptions on the persistence of SRB measures are needed to prove that $A^{(\varepsilon )}$ is an observable weak chimera.

## A Numerical Example of a Chaotic Weak Chimera with Trivial Symmetry

We now give an explicit example of a coupling function such that the dynamics of the product system (29) give rise to a chaotic weak chimera with trivial symmetry for $\varepsilon >0$ following the construction described in the previous section. In contrast to the examples in (Bick and Ashwin 2016), the main focus here is on the symmetries of the weak chimeras which we calculate explicitly.

**Fig. 1:**
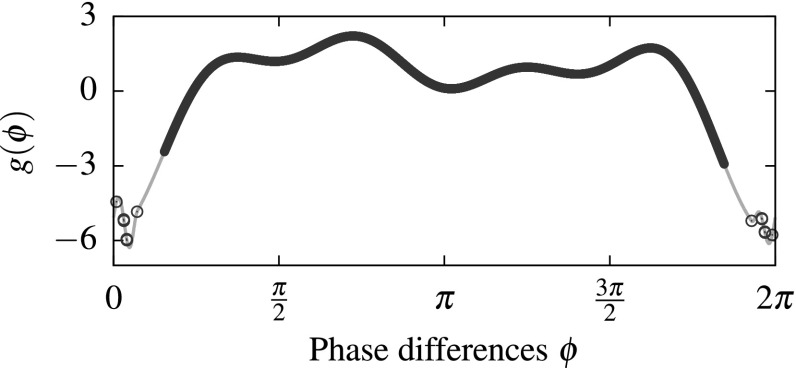
Attracting sets $A_{{\text {inc}}}$ and $A_{{\text {coh}}}$ of the dynamics given by (22) with coupling function $\hat{g}$ are coupling function separated. The coupling function $\hat{g}$ as defined in (33) is depicted by a *gray line*. The *values* of *g* on $\Xi (A_{{\text {inc}}})$ are indicated by *filled circles* and on $\Xi (A_{{\text {coh}}})$ by 12 *hollow circles*

Recall that the dynamics of (22) for $n=4$ oscillators give rise to chaotic attractors *A* with $\Sigma (A)=\left\{ {{\mathrm{id}}}\right\}$ (Bick et al. 2011; Bick 2012). Define

31 $$\begin{aligned} {g}(\phi ) = \sum _{r=0}^{4} c_r \cos \left( r\phi +\xi _r\right) \end{aligned}$$

with $c_1 = -2$, $c_2 = -2$, $c_3 = -1$, and $c_4 = -0.88$. For $\xi _1 = \eta _1$, $\xi _2 = -\eta _1$, $\xi _3 = \eta _1+\eta _2$, and $\xi _4 = \eta _1+\eta _2$ with $\eta _1=0.138$, $\eta _2=0.057511$ the dynamics of (22) with this particular choice of coupling function *g* give rise to a chaotic attracting set $A_{{\text {inc}}}\subset {\mathcal {C}}$ with positive maximal Lyapunov exponents and $\Sigma (A_{{\text {inc}}}) = \left\{ {{\mathrm{id}}}\right\}$. For $A_{{\text {inc}}}$, we have $\Xi (A_{{\text {inc}}})\subset [0.4, 2\pi -0.4]$ as shown in Fig. 1.

A suitable local perturbation of the coupling function *g* yields bistability between $A_{{\text {inc}}}$ and a relative equilibrium with trivial symmetry in the system defined by (22). Let

32 $$\begin{aligned} \tilde{g}(\phi ) = \sum _{r=6}^{24} a_r \cos (r\phi +\zeta _r) \end{aligned}$$

with parameters $a_r$, $\zeta _r$ as given in Appendix A. Moreover, define

$$\begin{aligned} \beta (x) := {\left\{ \begin{array}{ll}\exp \left( -\frac{1}{1-x^2}\right) &{}\quad \text {if} -1<x<1,\\ 0&{} \quad \text {otherwise}\end{array}\right. } \end{aligned}$$

and let $a\in {\mathbb {R}}$, $b \in (0, \pi )$ be parameters. Now, define $\beta _{ab}(\phi ) := a \beta \left( \frac{\phi }{b}\right)$ with $\phi$ taken modulo $2\pi$ with values in $(-\pi , \pi ]$ is a $2\pi$-periodic “bump function.” Fix $a = 2.5$, $b=0.25$. Define the $C^\infty$ function

33 $$\begin{aligned} \hat{g}:= g + \tilde{g}\beta _{ab}. \end{aligned}$$

We have $\hat{g}(\phi ) = g(\phi )$ for all $\phi \in [b, 2\pi -b]$. Since $\Xi (A_{{\text {inc}}})\subset [b, 2\pi -b]$, we have $Y^{(\hat{g})}(\varphi )=Y^{(g)}(\varphi )$ for all $\varphi \in A_{{\text {inc}}}$. Thus, $A_{{\text {inc}}}\subset {\mathcal {C}}$ is also a chaotic attracting set for the dynamics of (22) with coupling function $\hat{g}$. In addition, there is a stable relative periodic orbit $\varphi ^\star (t) \approx (t{\omega ^\star }, 0.0975+ t{\omega ^\star }, 0.1253+ t{\omega ^\star }, 0.2247+ t{\omega ^\star })$. For $A_{{\text {coh}}}=\left\{ \, \varphi ^\star (t)\,\left| \;t\ge 0\right. \right\}$ we have $\Xi (A_{{\text {coh}}})\subset [-0.3, 0.3]$. Therefore, the sets $A_{{\text {inc}}}$ and $A_{{\text {coh}}}$ are coupling function separated; see Fig. 1.

**Fig. 2:**
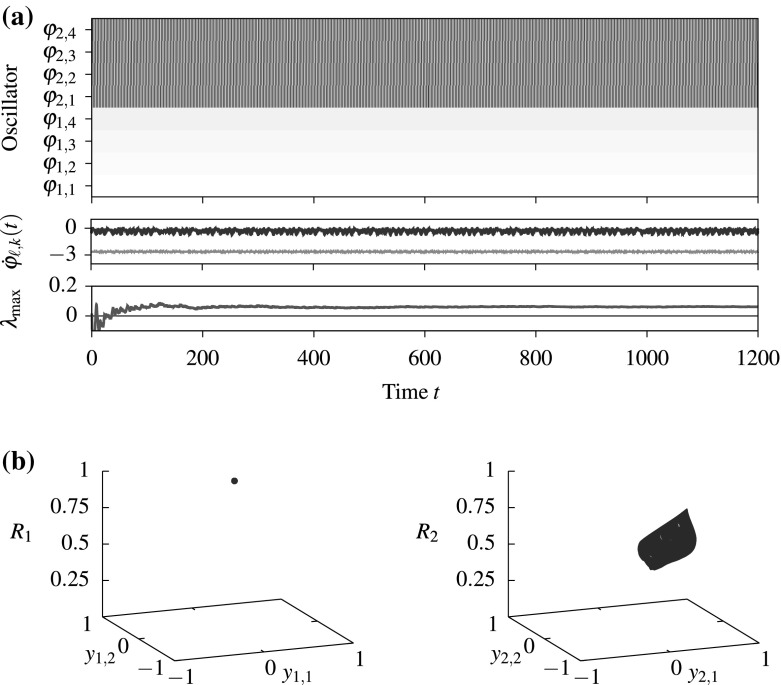
Chaotic weak chimeras with trivial setwise symmetries appear in the ${\mathbf {S}}_{4}\wr {\mathbf {S}}_{2}$-equivariant system (29) with two populations of $n=4$ oscillators for $\varepsilon =0.01$. **a** The phase evolution: The phase of the oscillators (periodic color scale, $\varphi _{\ell ,k}(t)=0$ in *black* and $\varphi _{\ell ,k}(t)=\pi$ in *white*) in a co-rotating frame at the speed of the first oscillator is shown at the top, the instantaneous frequencies $\dot{\varphi }_{\ell ,k}(t)$ in the middle and convergence of the maximal Lyapunov exponent at the bottom. **b** The dynamics on the attracting set for each population in the ${\mathbb {Z}}_4$-equivariant projections $y_\ell = (\sin (\varphi _{\ell ,3}-\varphi _{\ell ,1}), \sin (\varphi _{\ell ,4}-\varphi _{\ell ,2}))$ where ${\mathbb {Z}}_4$ are the permutations within populations that preserve the phase ordering, **a** phase evolution and **b** projected dynamics of each population

Now, consider two weakly coupled populations (29) of $n=4$ oscillators. Since $\Sigma (A_{{\text {coh}}})=\Sigma (A_{{\text {inc}}})=\left\{ {{\mathrm{id}}}\right\}$, we have that $\Sigma (A_{{\text {coh}}}\times A_{{\text {inc}}})=\left\{ {{\mathrm{id}}}\right\}$ for $\varepsilon =0$ and we expect dynamically invariant sets with trivial symmetry for small $\varepsilon >0$. We integrated system (29) numerically^3^ and calculated the maximal Lyapunov exponent from the variational equations. The attracting set $A^{(\varepsilon )}$ for $\varepsilon =0.01$ close to $A_{{\text {coh}}}\times A_{{\text {inc}}}$ with trivial setwise symmetries and positive maximal Lyapunov exponent is shown in Fig. 2; the absolute value of the local order parameter $R_\ell (t)=\big |\frac{1}{4}\sum _{j=1}^4\exp (i\varphi _{\ell ,j})\big |$ gives information about the synchronization of each population: It is equal to one if all oscillators within the populations are phase-synchronized.

**Fig. 3:**
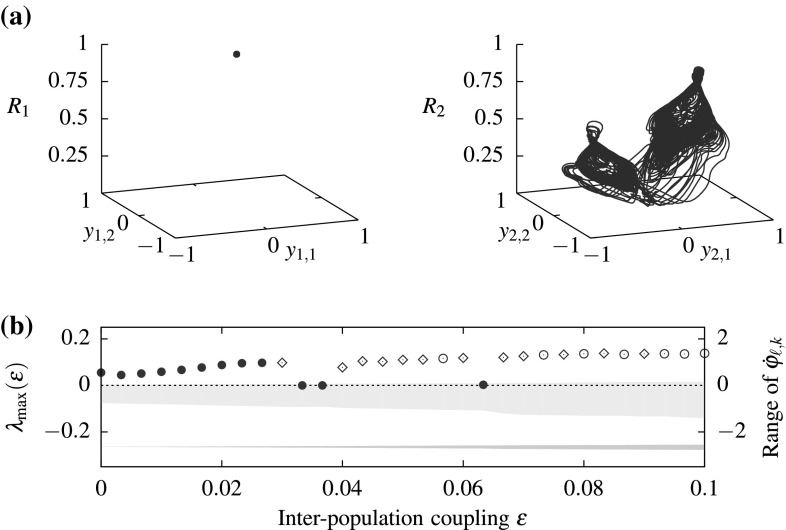
Increasing $\varepsilon$ yields chaotic weak chimeras that undergo symmetry increasing bifurcations. **a** A trajectory for $\varepsilon =0.1$ converging to an attractor $A^{(\varepsilon )}$ with $\Sigma (A^{(\varepsilon )})\ne \left\{ {{\mathrm{id}}}\right\}$. **b** Maximal Lyapnuov exponents obtained by integrating (29) from a fixed initial condition on $A^{(0)}$ for $T=2\times 10^5$ time units. The *marker* indicates the symmetry of the attractor $A^{(\varepsilon )}\subset {\mathbf {T}}^{2n}$: “$\bullet$” for $\Sigma (A^{(\varepsilon )})=\left\{ {{\mathrm{id}}}\right\}$, “$\diamond$” if $\Sigma (A^{(\varepsilon )})\mathop {=}\limits ^{?}\left\{ {{\mathrm{id}}}\right\}$, and “$\circ$” if $\Sigma (A^{(\varepsilon )})\ne \left\{ {{\mathrm{id}}}\right\}$. The *shaded regions* show the intervals $[\min _{k,t}\dot{\varphi }_{\ell ,k}(t), \max _{k,t}\dot{\varphi }_{\ell ,k}(t)]$ for $\ell =1$ (*dark gray*) and $\ell =2$ (*light gray*)—where these do not overlap, there is no frequency synchronization between the two populations and hence a weak chimera, **a** attractor in the $\mathbf{S}_{4}$-equivariant projection $y_{\ell }$, **b** maximal Lyapunov exponents and symmetries with varying $\varepsilon$

For increasing coupling parameter $\varepsilon$ (while keeping the initial condition fixed), the symmetries of the attracting chaotic weak chimeras $A^{(\varepsilon )}$ change; cf. Fig. 3. We integrated the system for $T=2\times 10^5$ time units to calculate both the maximal Lyapunov exponents and detect the presence of nontrivial symmetries. For $A^{(\varepsilon )}\subset {\mathcal {C}}^2$, we have to check for permutations of oscillators that preserve the ordering of the phases within each population to determine the symmetry of the attractor. To this end, we calculated the ergodic average $S_\ell (\varepsilon ) = \int _0^{{T}}\sin (\varphi _{\ell ,3}(t)-\varphi _{\ell ,1}(t)){\mathrm {d}}t$ along the trajectory which converges zero if $\Sigma (A^{(\varepsilon )})\ne \left\{ {{\mathrm{id}}}\right\}$. Note that if symmetric copies of attractors merge in a symmetry increasing bifurcation (Chossat and Golubitsky 1988), these ergodic averages may converge very slowly. Previous numerical investigations of the chaotic attractor in the uncoupled system (Bick 2012) showed that attractors with trivial symmetry are confined to one quadrant under the projection $y_\ell = (\sin (\varphi _{\ell ,3}-\varphi _{\ell ,1}), \sin (\varphi _{\ell ,4}-\varphi _{\ell ,2}))$. Thus, the number of quadrants $Q_\ell (\varepsilon )$ that the projected trajectory enters being greater than one indicates that a symmetry increasing bifurcation may have occurred; compare also Figs 2b and 3a. Consequently, we conclude $\Sigma (A^{\varepsilon })=\left\{ {{\mathrm{id}}}\right\}$ if $\left| S_2(\varepsilon )\right| >10^{-1}$—but we write $\Sigma (A^{\varepsilon })\mathop {=}\limits ^{?}\left\{ {{\mathrm{id}}}\right\}$ if $Q_2(\varepsilon )>1$ at the same time to indicate that a symmetry increasing bifurcation may have happened already—and $\Sigma (A^{\varepsilon })\ne \left\{ {{\mathrm{id}}}\right\}$ otherwise.

Further numerical investigation shows that there is multistability for $\varepsilon \ge 0$; the attracting sets $A^{(\varepsilon )}$ for $\varepsilon >0$ may coexist with other attracting solutions (not shown).

## Discussion

If a dynamical system has permutational symmetry $\Gamma$, what are the symmetry properties of the asymptotic angular frequencies which describe how trajectories wind around phase space? For the dynamical systems on ${\mathbb {C}}_\bullet ^n$ considered in Sect. 3, the asymptotic angular frequencies are given by averages of $\Gamma$-equivariant observables. This observation yields a natural reformulation of the notion of a weak chimera in terms of the isotropy of the vector of asymptotic angular frequencies. Our definition is not only compatible with the action of $\Gamma$ but also goes beyond phase oscillators in the weak coupling limit: It applies to more general oscillator models where chimera states have been reported (Sethia et al. 2013; Zakharova et al. 2014). With a rigorous definition in place, it would be desirable to prove the existence of weak chimeras in such systems and show that the dynamics observed are persistent phenomena. These ideas equally apply to more general spaces ${\mathbf {X}}$ with a symmetry group acting on it; here, asymptotic winding numbers of asymptotic cycles describe the rotation of a trajectory with respect to topological properties of ${\mathbf {X}}$ (Schwartzman 1957; Fried 1982; Walsh 1995). Precisifying the notion of a weak chimera for general topological spaces ${\mathbf {X}}$ with symmetry is beyond the scope of the current paper and will be addressed in future work.

Using coupling functions that give rise to relative equilibria with trivial symmetry, we showed that for symmetric phase oscillator systems that there are indeed weak chimeras that have symmetries in the frequencies that are not present in the solutions. This motivates some further symmetry-related questions. For example, what are the possible isotropy groups of the angular frequency vector for a $\Gamma$-equivariant system that do not arise from the symmetries of the solutions themselves? (These are obviously restricted to subgroups of the symmetry group.) Which symmetry increasing bifurcations happen as the inter-population coupling $\varepsilon$ is increased (Fig. 3)? While chaotic dynamics do persist up to $\varepsilon \approx 0.1$ [and for other choices of coupling function even up to $\varepsilon \approx 0.3$ (Bick and Ashwin 2016)], chaotic weak chimeras with trivial symmetry only persist for values of $\varepsilon$ close to zero. Thus, are there chaotic weak chimeras with trivial symmetry for “strongly coupled” populations of phase oscillators? Moreover, in general, there will be more than one ergodic invariant measure supported on a weak chimera. For each of these measures, we obtain asymptotic angular frequency vectors that potentially have different isotropy. While the set of all measures supported on the invariant set of interest (Jenkinson 2006) yields bounds of the asymptotic angular frequencies (see also Bick and Ashwin 2016), a more detailed understanding what the specific isotropy subgroups for the invariant measures are and how they bifurcate would be desirable.

It is also worth noting that asymptotic angular frequencies as averages and their isotropy may still be well defined if the permutational symmetry of the system is broken due to a (small) perturbation. However, care has to be taken to extend the notion of a weak chimera to nearly symmetric systems since symmetry breaking can have drastic effects on frequency synchronization (Ashwin et al. 2006b).

“Classical” chimera states were first observed on rings of nonlocally coupled phase oscillators (Kuramoto and Battogtokh 2002). A finite-dimensional approximation yields a dynamical system that is equivariant with respect to the action of the dihedral group (Ashwin and Swift 1992). Roughly speaking, classical chimeras on finite-dimensional rings are trajectories that show characteristic angular frequency synchronization for some finite time as they exhibit pseudo-random drift along the ring before converging to the fully synchronized state (Omel’chenko et al. 2010; Wolfrum and Omel’chenko 2011). These are not weak chimeras in the sense defined above. By contrast, initial conditions in the (dynamically invariant) fixed point spaces of a reflection symmetry yield symmetric solutions that eventually converge to the fully synchronized state (Omel’chenko 2015), resembling a transient weak chimera. Interestingly, the chaotic weak chimeras constructed here share an important feature with these “classical” chimera states: The isotropy of the angular frequency vector may be larger than the symmetry of the solution itself as the oscillators in the “coherent” region are never perfectly phase synchronized. Thus, clarifying the relationship between classical chimera states on rings and the symmetry of the system further—also with respect to the symmetries of the system that describes the continuum limit—provides exciting directions for future research.

## Appendix: A Trigonometric Polynomial Coupling Function

The Fourier coefficients of (32) in Sect. 6 are given by

$$\begin{aligned} a_6&=-0.676135392447403&\zeta _6&=0.846647746060342\\ a_8&=0.844660333390606&\zeta _8&=0.954985847962987 \\ a_{10}&=0.087624615584542&\zeta _{10}&= 0.212748482509925 \\ a_{12}&=-0.644961491438887&\zeta _{12}&= 0.025296512718163 \\ a_{14}&= -0.459724407978054&\zeta _{14}&= 0.180050952622569\\ a_{16}&= -1.175355598611419&\zeta _{16}&= 0.835173783095831 \\ a_{18}&=0.799302873723814&\zeta _{18}&= 0.850732311209280 \\ a_{20}&= -1.303832930713680&\zeta _{20}&= 0.863697094160152 \\ a_{22}&= 0.094742514998172&\zeta _{22}&= 0.355260772731067 \\ a_{24}&= -2.293749915528502&\zeta _{24}&= 0.463364388737488 \end{aligned}$$

and $a_r=0$, $\zeta _r=0$ for all other $r\in {\mathbb {N}}$.

## Footnotes

1 One can think of each complex variables $z_k$ representing phase and amplitude of an oscillator.

2 We obtain (22) by setting $H_{kj}=1$ for all *k*, *j* in (1).

3 Integration was carried out in MATLAB using the variable order scheme ode113 with a adaptive time step $\Delta t\le 10^{-1}$ subject to conservative relative and absolute error tolerances of $10^{-9}$ and $10^{-11}$, respectively.
